# Aligning intuition and theory: a novel approach to identifying the determinants of behaviours necessary to support implementation of evidence into practice

**DOI:** 10.1186/s13012-023-01284-1

**Published:** 2023-07-20

**Authors:** Natalie Taylor, Skye McKay, Janet C. Long, Clara Gaff, Kathryn North, Jeffrey Braithwaite, Jill J. Francis, Stephanie Best

**Affiliations:** 1 School of Population Health, Faculty of Medicine and Health, UNSW Sydney, High Street Kensington, Sydney, NSW 2052 Australia; 2 Australian Institute of Health Innovation, Macquarie University, Sydney, Australia; 3 Melbourne Genomics Health Alliance, University of Melbourne, Melbourne, Australia; 4 Australian Genomics, Murdoch Children’s Research Institute, Royal Children’s Hospital, Melbourne, Australia; 5 School of Health Sciences, University of Melbourne, Melbourne, Australia; 6 Department of Health Services Research, Peter MacCallum Cancer Centre, Melbourne, Australia; 7 Victorian Comprehensive Cancer Centre Alliance, Melbourne, Australia; 8 Sir Peter MacCallum Cancer Centre Department of Oncology, University of Melbourne, Melbourne, Australia

**Keywords:** Implementation science, Mechanism of action, Clinical genomics, Clinical practice change, Algorithm

## Abstract

**Background:**

Disentangling the interplay between experience-based intuition and theory-informed implementation is crucial for identifying the direct contribution theory can make for generating behaviour changes needed for successful evidence translation. In the context of ‘clinicogenomics’, a complex and rapidly evolving field demanding swift practice change, we aimed to (a) describe a combined clinician intuition- and theory-driven method for identifying determinants of and strategies for implementing clinicogenomics, and (b) articulate a structured approach to standardise hypothesised behavioural pathways and make potential underlying theory explicit.

**Methods:**

Interview data from 16 non-genetic medical specialists using genomics in practice identified three target behaviour areas across the testing process: (1) identifying patients, (2) test ordering and reporting, (3) communicating results. The Theoretical Domains Framework (TDF) was used to group barriers and facilitators to performing these actions. Barriers were grouped by distinct TDF domains, with ‘overarching’ TDF themes identified for overlapping barriers. Clinician intuitively-derived implementation strategies were matched with corresponding barriers, and retrospectively coded against behaviour change techniques (BCTs). Where no intuitive strategies were provided, theory-driven strategies were generated. An algorithm was developed and applied to articulate how implementation strategies address barriers to influence behaviour change.

**Results:**

Across all target behaviour areas, 32 identified barriers were coded across seven distinct TDF domains and eight overarching TDF themes. Within the 29 intuitive strategies, 21 BCTs were represented and used on 49 occasions to address 23 barriers. On 10 (20%) of these occasions, existing empirical links were found between BCTs and corresponding distinct TDF-coded barriers. Twenty additional theory-driven implementation strategies (using 19 BCTs on 31 occasions) were developed to address nine remaining barriers.

**Conclusion:**

Clinicians naturally generate their own solutions when implementing clinical interventions, and in this clinicogenomics example these intuitive strategies aligned with theoretical recommendations 20% of the time. We have matched intuitive strategies with theory-driven BCTs to make potential underlying theory explicit through proposed structured hypothesised causal pathways. Transparency and efficiency are enhanced, providing a novel method to identify determinants of implementation. Operationalising this approach to support the design of implementation strategies may optimise practice change in response to rapidly evolving scientific advances requiring swift translation into healthcare.

**Supplementary Information:**

The online version contains supplementary material available at 10.1186/s13012-023-01284-1.

Contributions to the literature This work has:

- Demonstrated the use of clinician intuition with evidence-based theory to generate contextually relevant implementation strategies;
- Identified that 20% of clinician intuitively-derived strategies aligned with theoretical behaviour change domains and corresponding BCTs that demonstrate evidence of mechanistic links;
- Generated standardised hypothesised behavioural pathways (with accompanying figures and an algorithm) to make underlying theory explicit, enhancing transparency and efficiency

## Background

The rapid, complex, and unpredictable nature of scientific advances is exceeding the ability of health systems to harness them. There are now over 35 million articles in PubMed, with 3000–5000 papers published every day [1]. Success will be dependent on capacities to continuously evolve to generate ideal conditions, systems and behaviours for successful implementation of research evidence into complex healthcare settings [2]. Rather, iterative attempts to apply complex interventions within existing clinical practice generate emergent routines with varying levels of suitability, efficiency, and sustainability. Recent developments in complex intervention research stress that improving theories and understanding of how interventions interact with their context and wider dynamic systems is an important goal to establishing intervention effectiveness [3]. Key activities that can contribute to achieving this goal include evidence based implementation practice [4]—deliberate efforts to increase impact and uptake of successfully tested interventions [3], and implementation science—the scientific study of methods and strategies that facilitate the uptake of evidence-based practice and research into regular use by practitioners and policymakers [5].

In addition to providing systematic approaches to planning and applying an implementation approach, using an evidence-based theory, model or framework can help to ensure that standardised approaches are taken to allow for accurate measurement, identification, replication, and refinement of the active ingredients behind implementation success [6–10]. For example, the Theoretical Domains Framework (TDF) encompasses 14 determinants and 84 component constructs of healthcare professional behaviour change (Table 1) [11, 12]. This framework can facilitate exploration of barriers and facilitators to implementing evidence-based behaviours [13] and provides a systematic, evidence-based pathway for implementation strategy design. Classification of barriers and facilitators according to the TDF can inform the selection of targeted behaviour change techniques (BCTs) empirically linked to theoretical determinant constructs [14, 15]. A standardised terminology exists which consolidates links between BCT definitions [9] and their mechanisms of action (MoAs) as represented through theoretical constructs (e.g., from the TDF) [11] from the existing evidence base [6]. If applied appropriately and recorded accurately, this can save precious time and resources for those attempting to solve similar problems across contexts, ultimately reducing research waste [16].

**Table 1:** The theoretical domains framework

Domain (definition)	Constructs
1. Knowledge (an awareness of the existence of something)	*Knowledge; procedural knowledge; knowledge of task environment*
2. Skills (an ability or proficiency acquired through practice)	*Skills; skills development; competence; ability; interpersonal skills; practice; skill assessment*
3. Social/professional role and identity (A coherent set of behaviours and displayed personal qualities of an individual in a social or work setting)	*Professional identity; professional role; social identity; identity; professional boundaries; professional confidence; group identity; leadership; organisational commitment*
4. Beliefs about capabilities (Acceptance of the truth, reality or validity about an ability, talent or facility that a person can put to constructive use)	*Self-confidence; perceived competence; self-efficacy; perceived behavioural control; beliefs; self-esteem; empowerment; professional confidence*
5. Optimism (the confidence that things will happen for the best or that desired goals will be attained)	*Optimism; pessimism; unrealistic optimism; identity*
6. Beliefs about consequences (acceptance of the truth, reality, or validity about outcomes of a behaviour in a given situation)	*Beliefs; outcome expectancies; characteristics of outcome expectancies; anticipated regret; consequents*
7. Reinforcement (increasing the probability of a response by arranging a dependent relationship, or contingency, between the response and a given stimulus)	*Rewards; incentives; punishment; consequents; reinforcement; contingencies; sanctions*
8. Intentions (a conscious decision to perform a behaviour or a resolve to act in a certain way)	*Stability of intentions; stages of change model; transtheoretical model and stages of change*
9. Goals (mental representations of outcomes or end states that an individual wants to achieve)	*Goals (distal/proximal); goal priority; goal/target setting; goals (autonomous/controlled); action planning; implementation intention*
10. Memory, attention, and decision processes (the ability to retain information, focus selectively on aspects of the environment and choose between two or more alternatives)	*Memory; attention; attention control; decision making; cognitive overload/tiredness*
11. Environmental context and resources (any circumstance of a person’s situation or environment that discourages or encourages the development of skills and abilities, independence, social competence and adaptive behaviour)	*Environmental stressors; resources/material resources; organisational culture/climate; salient events/critical incidents; person* × *environment interaction; barriers and facilitators*
12. Social influences (Those interpersonal processes that can cause individuals to change their thoughts, feelings, or behaviours)	*Social pressure; social norms; group conformity; social comparisons; group norms; social support; power; intergroup conflict; alienation; group identity; modelling*
13. Emotion (a complex reaction pattern, involving experiential, behavioural, and physiological elements, by which the individual attempts to deal with a personally significant matter or event)	*Fear; anxiety; affect; stress; depression; positive/negative affect; burn-out*
14. Behavioural regulation (anything aimed at managing or changing objectively observed or measured actions)	*Self-monitoring; breaking habit; action planning*

Table sourced from [11]: Validation of the theoretical domains framework for use in behaviour change and implementation research” by Cane, J., O’Connor, D. & Michie, S. 2012, *Implementation Science*, *7 (37)*. Copyright © 2012, Cane et al.; *licensee BioMed Central Lt*

The ideals of rigorous theory-driven approaches to implementation, however, have often been met with significant yet unpredictable contextual and interpersonal complexities, leading to *overlapping* barriers [17–19]. These overlapping barriers must be accounted for and incorporated but are difficult to manage and measure [20–22]. Such complexities present challenges for practitioners and researchers regarding ‘staying true’ to a particular theoretical approach (i.e., theoretical fidelity) [23–25] whilst accounting for and responding flexibly to healthcare professional, patient, and system needs [22, 26–33]. Challenges are further exacerbated when the intricacies of theory application can be inaccessible to non-experts [7], and slow, relative to demands for rapid evidence translation [22, 33, 34]. Although theory-driven stakeholder co-design methodology is evolving [35–37], theory-based approaches to elicit key barriers to implementation of a particular intervention and inform implementation strategy design can lead to stakeholders intuitively identifying ‘on the spot’ solutions [38]. These solutions may or may not align with theoretical recommendations, and stakeholders may also decide to enact them immediately, despite deviation from the implementation protocol [22, 26, 39]. Although alternative approaches and adaptations may well be effective, given the tacit knowledge and experience of clinicians, these solutions are often not recorded, making it difficult to identify the extent to which (a) deviation from the theorised core functions has occurred, and (b) they are effective.

One area of exploration is the role of healthcare professional experience-based intuition in the identification of barriers and solutions to implementation, and the extent to which this intuition aligns with theory-driven recommendations [16, 21, 38, 40–42]. Enhancing understanding about the alignment of experience-based intuition and theory can tell us more about where healthcare professionals generate relevant implementation strategies (e.g., education/training) to address identified barriers (e.g., knowledge/skills) without the need for in-depth use of theory. It can also signal where theory is most needed (e.g., to address more complex barriers such as social influences or emotion) and can be best utilised—potentially making the development of theory-guided implementation strategies more efficient. Furthermore, recording and coding intuitively derived implementation strategies against theory can allow for the study of effects and contribute to the evidence-base for establishing and explaining the mechanistic links between strategies that lead to clinical practice change [38, 42]. This in turn could support adjustments to theory when theoretical predictions and empirical observations are inconsistent [43].

Clinicogenomics—using the entire genome of a patient to diagnose diseases or adjust medications exclusively for that patient [44]—is a rapidly evolving field and is already demanding swift clinical practice change at multiple levels as testing in healthcare becomes a reality [45–49]. During 2014–2019, 29 early adopter health system ‘flagship’ demonstration projects across Australia were using clinicogenomics as part of nested research studies sponsored by the Australian Genomics and Melbourne Genomics Health Alliance programmes [50, 51]. Together these alliances have placed emphasis on understanding, from an organisation level and clinical practice perspective, how genomic testing can be implemented in healthcare. As a result we have studied clinician emergent and self-organising behaviours (i.e., communal behaviours which create order through interactions) during the implementation of genomics into practice [47]; identified successful emergent behaviours and practice gaps [47, 52]; and synthesised this information using a theoretical framework [53, 54]. The insights of these early adopters are crucial for enhancing our understanding of the contribution of clinician intuition and theory for identifying determinants of and strategies for implementing clinicogenomics.

## Aim

This paper aims to (a) describe a combined clinician intuition and theory-driven approach to identifying determinants of and strategies for implementing clinicogenomics, and (b) articulate a structured approach to standardise hypothesised behavioural pathways and make potential underlying theory explicit. Our objectives were to.

1. Identify and code distinct barriers to implementation according to the TDF and group overlapping barriers into overarching TDF themes;
2. Map implementation strategies intuitively generated by clinicians to overcome barriers to implementation with evidence-based BCT definitions;
3. Identify the extent to which intuitive strategies align with theoretical behaviour change domains and corresponding BCTs that demonstrate evidence of mechanistic links;
4. Use TDF-BCT mechanistic links evidence to develop implementation strategies to overcome remaining TDF-matched barriers;
5. Develop an algorithm to articulate a structured approach to standardise hypothesised behavioural pathways.

## Methods

### Context

The work described here amalgamates early results of a Type 1 Hybrid study design as part of the Australian Genomics and Melbourne Genomics programmes of research, described in detail elsewhere [2]. To summarise, demonstration projects across 29 disease conditions integrating genomics into clinical settings have been studied to understand emergent and self-organising behaviours amongst inter-related actors and processes. Interview data from 32 participants (16 non-genetic medical specialists and 16 organisational management level professionals) involved in developing the genomics clinical practice systems and approaches across five flagships were synthesised to generate TDF-based barriers and facilitators to undertaking three key tasks (i.e., target behaviour areas: TBAs) crucial for the implementation of genomics [(1) ensuring appropriate patients are selected for genomic testing, (2) requesting testing and interpreting the data, and (3) communicating results to patients] [2]. Mixed methods were then used to conduct 16 additional process map guided TDF-informed interviews [55] with non-genetic medical specialists and identify the psychosocial and environmental determinants of change across the three target behaviour areas (TBAs) [2, 53]. This study reports on methodological approaches to describe a combined intuition- and theory-driven method for identifying determinants of and strategies for implementing clinicogenomics.

### Study design

We conducted an in-depth TDF-driven retrospective mapping exercise (implementation of clinicogenomics barriers and facilitators mapped to TDF domains and BCTs) using principles of implementation mapping (i.e., choose theoretical methods and select or design implementation strategies that preserve the parameters for clinical effectiveness and fit with the target population, culture, and context) [56–58]. We used this approach to synthesise findings from TDF-informed semi-structured interviews with non-genetic medical specialists, who identified factors affecting the implementation of genomics [53] and generated intuitive implementation strategies.

### Participants and recruitment

Following research ethical approval (Melbourne Health HREC: HREC/13/MH/326) and governance from participating organisations, interviews were undertaken with non-genetic medical specialists currently working in the field of genomics with either Australian Genomics or Melbourne Genomics. Recruitment details are provided elsewhere [53].

### Data collection and procedure

Our starting point was the initial synthesis undertaken on the 16 process map guided interview transcripts with non-genetic medical specialists (neurology = 4; cardiology = 1; nephrology = 6; immunology = 2; oncology = 3; including some with leadership roles = 7) [53, 55]. This work coded barriers and facilitators according to the TDF across the three specific TBAs along the genomics clinical pathway [2] to identify what factors facilitate or hinder the implementation of genomics into clinical practice by non-genetic specialists. Facilitators that were identified as implementation strategies suggested by participants (as currently utilised or potential strategies—hereon in referred to as intuitively derived or intuitive strategies) were also matched to barriers that they could directly address.

**Fig. 1:**
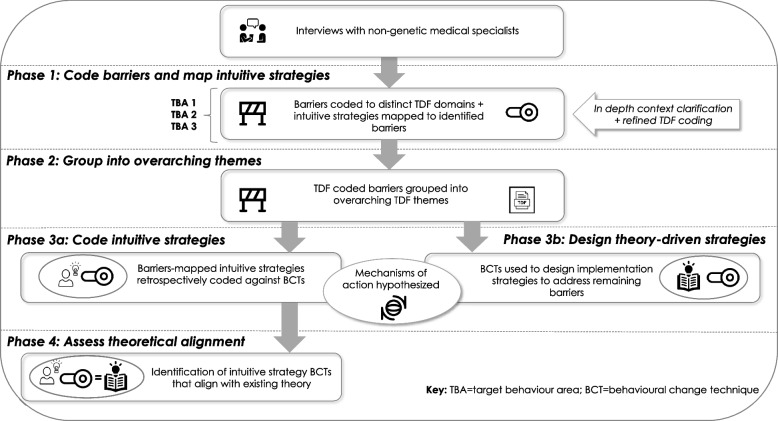
Overview of data synthesis process

Taking these systematically coded barriers and intuitive strategies, we commenced our four-phase mapping approach to data synthesis (Fig. 1): (1) in-depth context clarification and TDF construct coding for the identified barriers and intuitive strategies; (2) grouping of overlapping barriers according to overarching TDF themes; (3a) coding intuitive strategies against BCTs [9]; (3b) designing theory-driven implementation strategies using BCTs [8], and (4) assessing alignment of intuitive strategies and theory [6, 8].

### Data synthesis

#### Phase 1—in-depth context clarification and construct coding for identified barriers and intuitive strategies

Two authors (SB and NT) held 3 × 4-h meetings to work through the specific context of each barrier to clarify understanding, and justify the previously identified theoretical links between barriers, TDF domains, and intuitive strategy mapping to relevant corresponding barriers. These in-depth discussions revealed the need to refine some initial TDF domain categorisation and mapped intuitive strategies for which rationales were documented, accounting for the need for practical application in a way that preserves the parameters for clinical effectiveness and fits with the target population, culture, and context [58].

#### Phase 2—grouping of barriers according to overarching TDF themes

Given the recognisable relationships and interdependencies between *distinct* TDF-coded barriers that emerged through the analysis and further in-depth context clarification discussions, overlapping barriers were also grouped according to an *overarching* theme which encompassed several TDF domains (hereon in referred to as *overarching TDF theme*). This allocation of an overarching theme was important because it was often considered as a driver of or influence on barriers that were related to one another at a granular level, and indicated the need to consider barriers together, in context, when considering implementation strategy development.

#### Phase 3a—coding intuitive strategies against BCTs

**Fig. 2:**
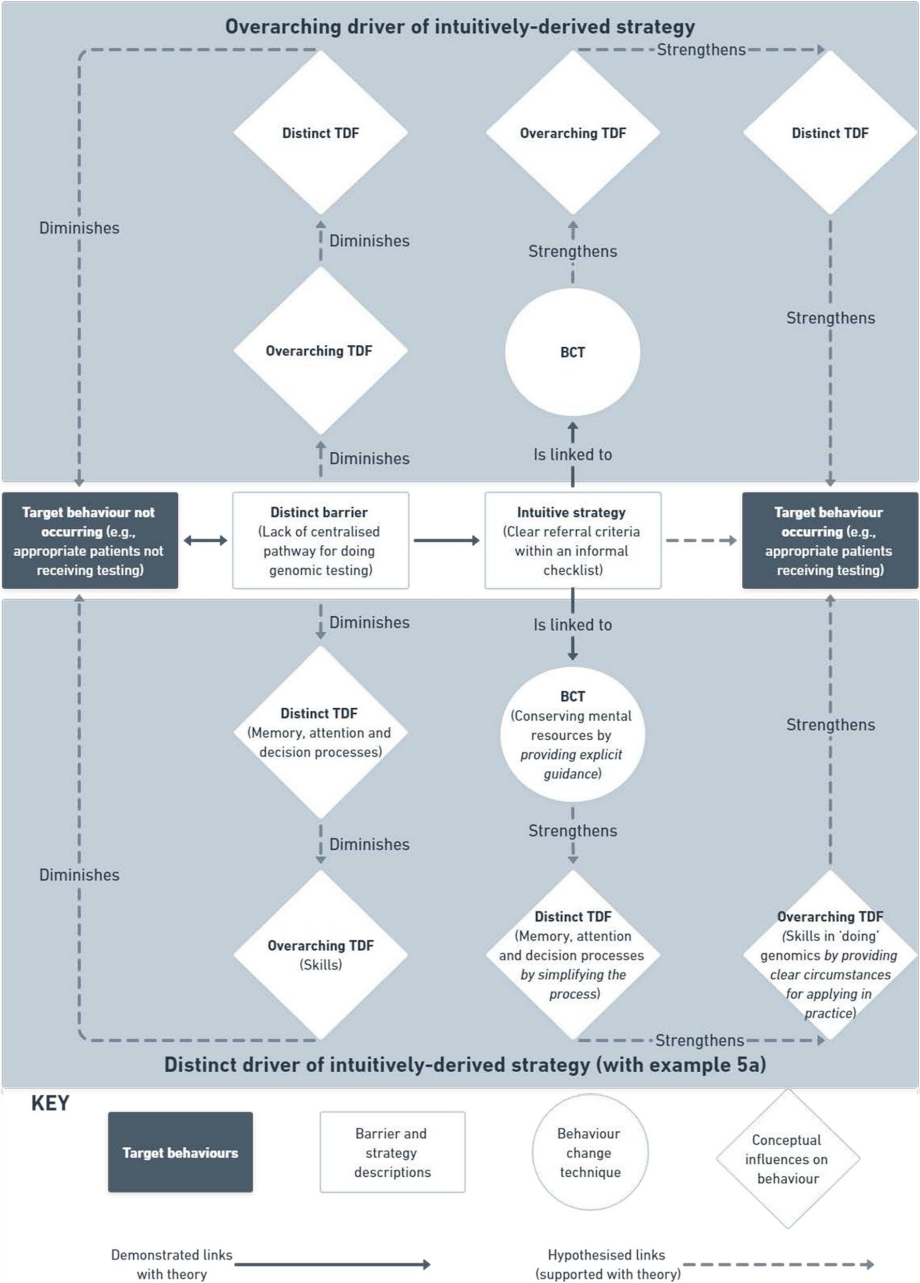
Intuitively derived strategies with overarching and distinct TDF driver pathways

The aim of this phase was to determine any instance a BCT was identified in an intuitively-derived strategy (noting each strategy can include multiple BCTs, and one BCT can be present in multiple strategies)—and are hereon in referred to as ‘occasions’ [38] (i.e., one occasion is equal to an instance whereby a BCT was identified in an intuitively derived strategy). In a series of 3 × 3-h meetings (NT, SB, JL), barrier-mapped intuitive strategies with sufficient description available directly from the dataset were retrospectively coded against evidence-based BCTs using the most up to date BCT definitions (see Additional file 1) [8, 9]. For those intuitive strategies without sufficient description, the research team further unpicked the context of the barriers they were intended to address, expounding the intuitive strategy to the extent it could be coded as a BCT. This was achieved mainly through probing the interviewer (SB) to understand the nuances of the described barriers. Guidance from the online BCT-TDF mapping tool [8] was next used to assess the extent to which these intuitive strategies aligned with the most up to date theoretical guidance for BCT selection. Theoretically underpinned links were then formally documented using a structured format (see Fig. 2 and example a in Figure 5) to hypothesise the MoAs for changes to barrier-specific, and subsequently, overarching TDF themes, as a result of intuitively-derived strategies retrospectively coded against BCTs [8].

As an example,

The construct of A and distinct TDF domain B were selected because the clinicians were *doing C behaviour*. The *intuitive strategy* of D is linked to the BCT E *because it will do F* to change their G (*distinct* TDF domain) by increasing H (*overarching* TDF theme) *because of X* (explanation).

In instances whereby the overarching TDF theme to which a barrier had been coded was classified as the initial MoA, the algorithm was amended such that changes to an overarching barrier leads to changes in a distinct barrier to eventually influence behaviour change:

The construct of A and distinct TDF domain B were selected because the clinicians were *doing C behaviour*. The *intuitive strategy* of D is linked to the BCT E *because it will do F* to change their G (*overarching* TDF theme) by increasing H (*distinct* TDF domain) *because of X* (explanation).

### Phase 3b—designing theory-driven strategies using BCTs

**Fig. 3:**
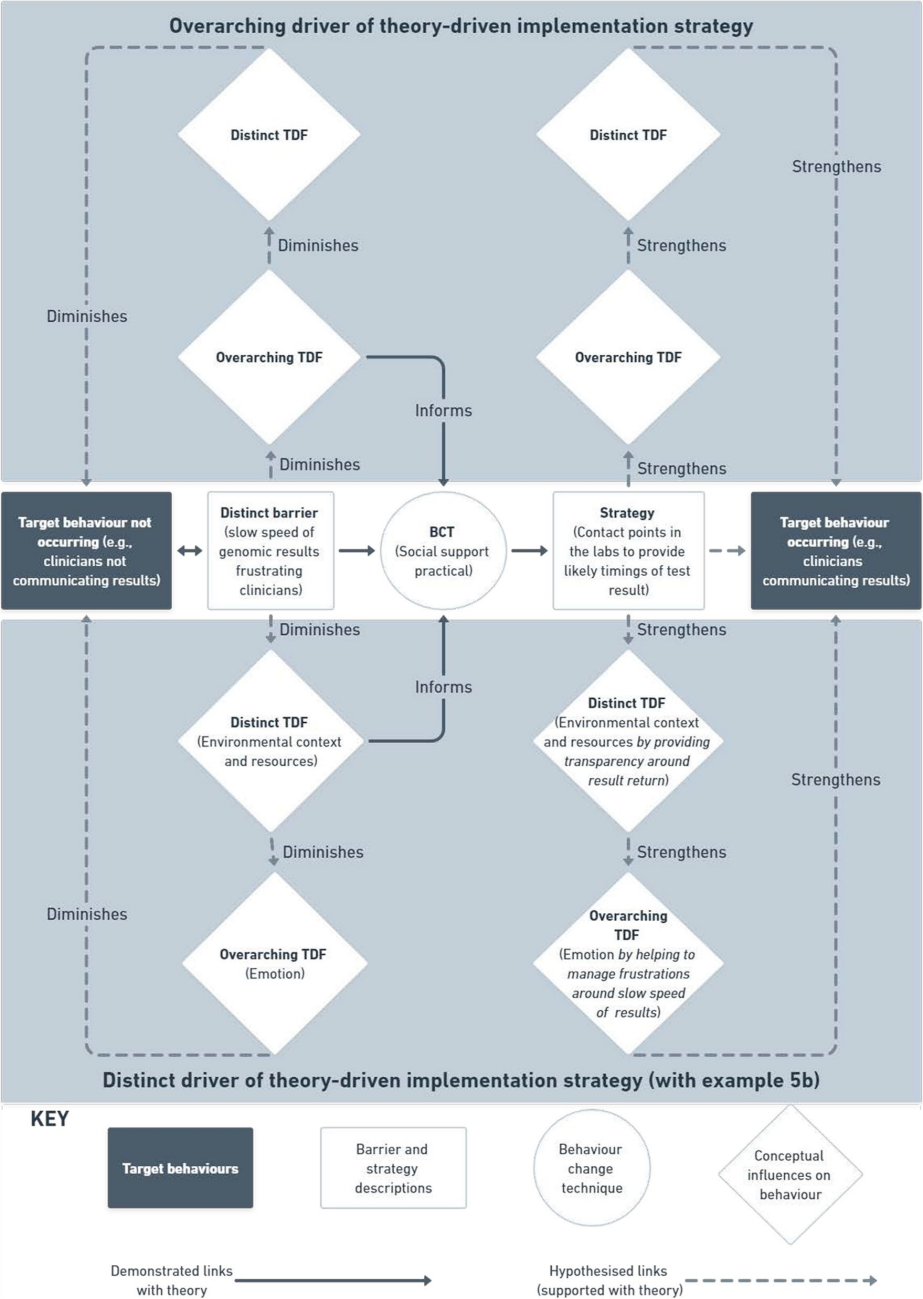
Theory-driven strategies with overarching and distinct TDF driver pathways

In a final set of 3 × 4-h meetings with SB and NT, implementation strategies were designed to address all remaining distinct level barriers (i.e., those without a mapped intuitively-derived strategy) through the use of only those BCTs with evidence of mechanistic links with TDF domains [8]. In this phase, we applied a two-tiered approach; that is, we initially mapped relevant BCTs to the distinct barrier—with the assumption that these then lead to improvements in the overarching barrier—and developed practical implementation strategies. In instances where BCTs were not helping to generate appropriate strategies to address the barrier-specific problem, we addressed the overarching barrier directly (using evidence-based BCTs mapped to the overarching barrier). Using the structured format outlined in Fig. 3 and example b in Figure 5, theoretically underpinned links were formally documented to hypothesise the MoAs for changes to barrier-specific, and subsequently, overarching TDF themes, based on using theory to design implementation strategies.

As an example:

The construct of A and distinct TDF domain B were selected because the clinicians *were doing C behaviour*. The *strategy* of D is derived from the BCT E *because it will do* F—this should reduce their G (*distinct* TDF domain) and change their H (*overarching* TDF theme) *because of X* (explanation).

In instances whereby the overarching TDF theme was classified as the initial MoA, the algorithm was amended such that changes to an overarching barrier leads to changes in a distinct barrier to eventually influence behaviour change:

The construct of A and distinct TDF domain B were selected because the clinicians were *doing C behaviour*. The *strategy* of D is derived from the BCT E because *it will do F*—this should reduce their G (*overarching* TDF theme) and change their H (*distinct* TDF domain) *because of  X *(explanation).

### Phase 4—assessing alignment of intuitive strategies and theory

In line with previously reported methods [38], a counting exercise was undertaken to assess the number of barriers and intuitively derived barrier-matched strategies. The number of intuitive strategies that aligned with BCTs demonstrating mechanistic links with the associated TDF domains, according to the Theory and Techniques Tool [8], were counted next to provide a proportion of alignment.

## Results

**Fig. 4:**
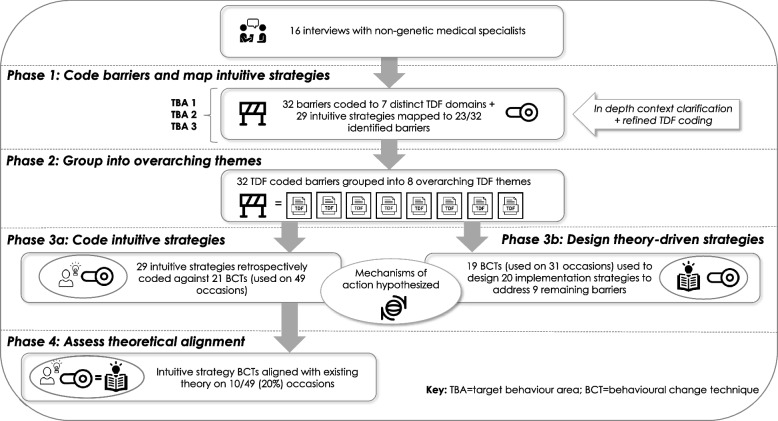
Summary of results of data synthesis

A total of 32 distinct barriers (20, 7, and 5 across TBAs 1–3, respectively) and 29 intuitive strategies (20, 4, and 5 across TBAs 1–3, respectively) were identified through the initial data analysis phase. The barriers were deductively coded according to distinct TDF domains [13], and through in-depth discussion, coding was refined, constructs were allocated, and overarching TDF themes were assigned to groups of barriers that were interrelated (Fig. 4).

### Phase 1—in-depth context clarification and construct coding for identified barriers and intuitive strategies

In-depth context clarification revealed crucial insights from the lead interviewer. This allowed for drilling down barriers and facilitators to the construct level, ensured confidence amongst the group that domains assigned to barriers were appropriate, and in instances where there was some uncertainty, provided an opportunity to unpick coding in relation to the context and make amendments where necessary. The need to change TDF domains to which barriers had been coded occurred in one case for TBA1 (ensuring appropriate patients receive testing) and in one case for TBA2 (requesting testing and interpreting the data). For example, a lay description of a barrier: ‘not trained to counsel’ was originally coded under the ‘knowledge’ domain. However, context clarification discussion records indicated: “clinicians talked about this passionately—they weren't trained to counsel people about genomic testing. Re-thinking and wondering whether skills is a better TDF fit”, and so this barrier was recoded to ‘skills’ (see Additional file 2).

A total of 32 barriers were coded into distinct TDF domains and constructs, with seven domains represented in total and coded between one (e.g., goals) and eight (knowledge) times across each TBA. Sixteen TDF constructs were coded between one (e.g., goal priority, professional boundaries) and four (e.g., skill development, decision making) times across each TBA (Additional file 3). Across all TBAs, there was some overlap of TDF domains (e.g., environmental context and resources, knowledge). TBA1 (ensuring appropriate patients receive testing) produced the largest number of barriers (*n* = 20), range of TDF domains (*n* = 6), and constructs (*n* = 13).

### Phase 2—grouping of barriers according to overarching TDF themes

A total of 13 overarching TDF themes were generated (TBA1 = 7, TBA2 = 4, TBA3 = 2), each of which encompassed between 1 and 5 individual interrelated barriers. As an example, barriers related to TBA3 (communicating results to patients) such as ‘evolving field’ (TDF = knowledge), ‘working in isolation’ (TDF = social influences), and ‘speed of results’ (TDF = environmental context and resources) were coded under a general theme of ‘feeling comfortable with communicating results’, with the corresponding overarching TDF theme being ‘Emotion’.

### Phase 3a—coding intuitive strategies against BCTs

A total of 21 BCTs were represented within the 29 intuitive strategies identified across TBAs 1–3 (see Additional file 2), which were found to be used on 49 occasions (i.e., each instance a BCT was identified within an intuitive strategy) [38].

Of the 21 BCTs represented, across all three TBAs, the most frequently coded were ‘conserving mental resources’ (represented in 7 intuitive strategies), followed by ‘social support: practical’ (represented in 6 intuitive strategies), and ‘credible source’ (represented in five intuitive strategies). The largest number of intuitive strategies (*n* = 20) was produced to address TBA1, represented by 14 different BCTs coded between one (e.g., ‘graded task’) and six (e.g., ‘conserving mental resources’) times.

**Fig. 5:**
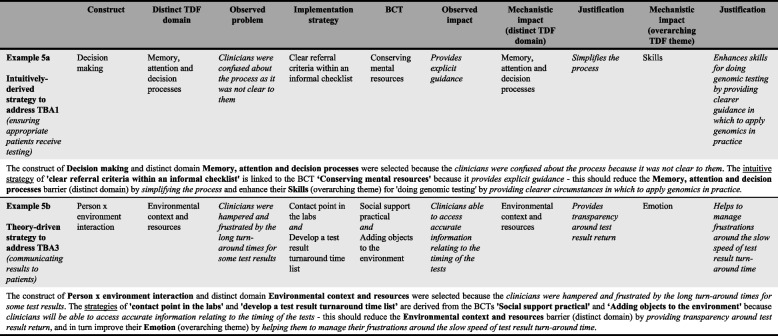
Application of algorithm across target behaviour areas

*Prior to theory-alignment assessment* from the existing evidence-base, using the algorithm, we hypothesised the MoA for changes to barrier-specific, and subsequently, overarching TDF themes, as a result of intuitively derived strategies retrospectively coded against BCTs. See Figs. 2 and 5 for a visual and algorithm-based example of an intuitively derived strategy to address TBA1 (ensuring appropriate patients receive testing) (example 5a).

### Phase 3b—designing theory-driven strategies using BCTs

A total of 20 original implementation strategies (*n* = 9, *n* = 7, and *n* = 4 across TBAs 1–3, respectively) were developed to address the nine remaining barriers that were not mapped to any suggested intuitive strategies. Strategies were designed using combinations of 19 BCTs (on 31 occasions) that have previously demonstrated mechanistic links with either the distinct TDF domains (*n* = 15), overarching TDF themes (*n* = 3), or both (*n* = *2*) in instances where the distinct and overarching codes are the same(6, 8) (Additional file 2). See Figs. 3 and 5 for a visual and algorithm-based example of a theory-driven strategy designed to address TBA3 (communicating results to patients) (example 5b).

Of the 19 BCTs, across all three TBAs, the most frequently coded were ‘adding objects to the environment’ and ‘social support: practical’ (coded four times each), followed by ‘restructuring the physical environment’ (coded three times). Table 2 presents a summary of the distinct barrier and overarching TDF coding for each TBA, as well as the number of barrier-mapped intuitively derived and theory-driven strategies designed for each.

**Table 2:** Summary of TBA barrier coding and intuitive and theory-driven implementation strategies

	No. distinct barriers	Distinct TDF domains (no. times coded)	No. overarching themes	Overarching TDF themes (no. times coded)	Implementation strategy summary
TBA1 *“Ensuring appropriate patients receive testing”*	20	• Skills (6) • Knowledge (4) • Environmental context and resources (4) • Memory, attention and decision processes (3) • Professional role and identity (2) • Goals (1)	7	• Beliefs about capabilities (2) • Skills (2) • Social influences (1) • Knowledge (1) • Beliefs about consequences (1)	• 20 intuitive strategies, addressing 16 distinct barriers (14 BCTs used on 31 occasions) • 9 theory-driven strategies, addressing 4 distinct barriers (11 BCTs used on 15 occasions)
TBA2 *“Requesting testing and interpreting the data”*	7	• Knowledge (3) • Professional role and identity (2) • Social influences (1) • Environmental context and resources (1)	4	• Intentions (2) • Social influence (1) • Professional role and identity (1)	• 4 intuitive strategies, addressing 4 distinct barriers (7 BCTs used on 12 occasions) • 7 theory-driven strategies, addressing 3 distinct barriers (7 BCTs used on 8 occasions)
TBA3 *“Communicating results to patients”*	5	• Environmental context and resources (2) • Memory, attention and decision processes (1) • Knowledge (1) • Social influences (1)	2	• Beliefs about capabilities (1) • Emotion (1)	• 5 intuitive strategies, addressing 3 distinct barriers (5 BCTs used on 6 occasions) • 4 theory-driven strategies, addressing 2 distinct barriers (6 BCTs used on 8 occasions)
Total	32	• Knowledge (8) • Environmental context and resources (7) • Skills (6) • Memory, attention and decision processes (4) • Professional role and identity (4) • Social influences (2) • Goals (1)	13	• Beliefs about capabilities (3) • Skills (2) • Social influences (2) • Intentions (2) • Beliefs about consequences (1) • Professional role and identity (1) • Knowledge (1) • Emotion (1)	• 29 intuitive strategies, addressing 23 distinct barriers (21 BCTs used on 49 occasions) • 20 theory-driven strategies, addressing 9 distinct barriers (19 BCTs used on 31 occasions)

#### Phase 4—assessing alignment of intuitive strategies and theory

Table 3 presents a summary of the alignment of intuitively-derived strategies and theory. After cross-referencing against the Theory and Techniques Tool [8], across all three TBAs, we found that of the 49 occasions in which BCTs were represented in intuitive strategies, 10 (20%) aligned with a corresponding *distinct* TDF domain coded barrier that has previously demonstrated statistically significant mechanistic links (i.e., theoretical alignment agreed upon by expert consensus AND associations in the implementation strategy literature synthesis). For example, TBA1 = 6/31 (19%)—‘conserving mental resources’ (TDF domain = memory, attention, and decision processes; used on two occasions), ‘behavioural practice/rehearsal’ (TDF domain = skills), and ‘social support (practical)’ (TDF domain = environmental context and resources; used on three occasions). Across all TBAs, there were 17/49 (35%) occasions where intuitive strategy BCTs aligned with a corresponding distinct *or* overarching TDF coded barrier. For example, TBA1 = 11/31 (35%)—in addition to the six distinct links described above included ‘demonstration of the behaviour’ (TDF domain = beliefs about capabilities), ‘salience of consequences’ (TDF domain = beliefs about consequences), ‘behavioural practice/rehearsal’ (TDF domain = beliefs about capabilities), ‘problem solving’ (TDF domain = beliefs about capabilities), and ‘graded task’ (TDF domain = beliefs about capabilities).

**Table 3:** Alignment of distinct and overarching barriers and intuitive strategies with theoretical recommendations

	Distinct barrier and intuitive strategy alignment with theoretical recommendations					
	Align with theory		Non-links		Inconclusive	No evidence
TBA1	6/31 (19%)	• ‘Conserving mental resources’—memory, attention, and decision processes; used on two occasions • ‘Behavioural practice/rehearsal’—skills • ‘Social support (practical)’ —environmental context and resources; used on three occasions	3/31 (10%)	• ‘Prompts and cues’—knowledge • ‘Conserving mental resources’—professional role and identity; used on two occasions	5/31 (16%)	17/31 (55%)
TBA2	2/12 (17%)	• ‘Information about social and environmental consequences’—knowledge • ‘Social support (unspecified)’ —social influences	2/12 (17%)	• ‘Problem solving’—professional role and identity • ‘Monitoring of outcomes of behaviour without feedback’—professional role and identity	1/12 (8%)	7/12 (58%)
TBA3	2/6 (33%)	• ‘Conserving mental resources’—memory, attention, and decision processes • ‘Social support (unspecified)’—social influences	1/6 (17%)	• ‘Action planning’—knowledge	0/6 (0%)	3/6 (50%)
Total^a^	10/49 (20%)		6/49 (12%)		6/49 (12%)	27/49 (55%)
	Distinct/overarching barrier and intuitive strategy alignment with theoretical recommendations					
	Align with theory		Non-links		Inconclusive	No evidence
TBA1	11/31 (35%)	• ‘Conserving mental resources’—memory, attention, and decision processes; used on two occasions • ‘Behavioural practice/rehearsal’—skills • ‘Social support (practical)’—environmental context and resources; used on three occasions • ‘Demonstration of the behaviour’—beliefs about capabilities • ‘Salience of consequences’—beliefs about consequences • ‘Behavioural practice/rehearsal’—beliefs about capabilities • ‘Problem solving’—beliefs about capabilities • ‘Graded task’—beliefs about capabilities	3/31 (10%)	• ‘Prompts and cues’—knowledge • ‘Conserving mental resources’—professional role and identity; used on two occasions	5/31 (16%)	12/31 (39%)
TBA2	4/12 (33%)	• ‘Information about social and environmental consequences’—knowledge • ‘Social support (unspecified)’ —social influences • ‘Information about others approval’—social influences • ‘Social comparison’—social influences	4/12 (33%)	• ‘Problem solving’—professional role and identity; used on two occasions • ‘Monitoring of outcomes of behaviour without feedback’—professional role and identity • ‘Problem solving’—social influences	2/12 (17%)	2/12 (17%)
TBA3	2/6 (33%)	• ‘Conserving mental resources’—memory, attention, and decision processes • ‘Social support (unspecified)’ —social influences	1/6 (17%)	• ‘Action planning’—knowledge	0/6 (0%)	3/6 (50%)
Total	17/49 (35%)		8/49 (16%)		7/49 (14%)	17/49 (35%)

^a^Decimal rounding down provides a total of 99%

For distinct barriers, five of the 21 intuitive strategy BCTs were found to be ‘non-links’ (e.g., BCT-MoA link absent in literature synthesis AND experts in consensus study agreed there was no theoretical link), and were used on 6/49 (12%) of occasions. These ‘non-links’ occurred in; TBA1: ‘prompts and cues’ (TDF domain = knowledge), ‘conserving mental resources’ (TDF domain = professional role and identity; used on two occasions); TBA2: ‘problem solving’ and ‘monitoring of outcomes of behaviour without feedback’ (TDF domain = professional role and identity); and TBA3: ‘action planning’ (TDF domain = knowledge). All these occasions were in the context of intuitive strategies with multiple BCTs coded (see Additional file 2), although none of the accompanying BCTs had evidence of mechanistic links. Six BCTs were found to be ‘non-links’ for distinct *or* overarching TDF coded barriers used on 8/49 (16%) of occasions. In addition to the previously listed distinct barrier ‘non-links’, these occurred in TBA2 and included ‘problem solving’ (TDF domain = professional role and identity; used on two occasions), ‘monitoring of outcomes of behaviour without feedback’ (TDF domain = professional role and identity), and ‘problem solving’ (TDF domain = social influences).

Some of the BCT links to theory were found to be inconclusive. For the distinct barriers, there were 6 (12%) occasions where intuitive strategy BCTs were found to be inconclusive and 7 (14%) for the distinct *or* overarching barriers. The remaining BCTs had either an absence of evidence to draw conclusions about mechanistic links for distinct barriers [remaining BCTs used on 27/49 (55%) occasions], or distinct *or* overarching barriers [remaining BCTs used on 17/49 (35%) occasions]. Additional file 3 provides levels of evidence and details for mechanistic links for all 49 occasions, as derived from the Theory and Techniques Tool.

Findings are reported in line with the TIDieR template for intervention description and replication (TIDieR) checklist and guide (Additional file 4).

## Discussion

We sought to use combined clinician experience-based intuition and theory-driven approaches to support the translation of genomics into the Australian healthcare system through the design of implementation strategies to address key barriers across three TBAs. In line with recommendations [3, 7], our theoretical framework (the TDF) was specified in advance to support the design of strategies. In addition, we aimed to identify existing intuitively-derived strategies or ‘on the spot’ recommendations for overcoming barriers and discovered that 20% were found to align with theoretical recommendations. Alongside this approach, we developed a novel algorithm and supporting diagrammatic theoretical pathways to standardise and aid transparency about hypothesised key steps in attempting to address TDF-specified barriers via strategies comprising BCTs, and the associated mechanistic links. Highlighting the unavoidable complexity of barriers and enhancing transparency of how these instances have been managed, we not only reported these algorithms for distinct barriers, but also incorporated overarching TDF themes to illustrate the relationships between distinct barriers and other antecedents or consequences. Whilst coding to TDF constructs that are grouped together in each domain has not typically been applied in the past [13, 15], we found discussing constructs was helpful for context clarification and was informative over and above domain-level coding for selecting BCTs.

The prominence of distinct TDF domains varied across each TBA (e.g., TDF domain ‘skills’ was represented six times across TBA1 but did not feature in TBA2 or TBA3, whereas ‘environmental context and resources’ was represented between one and four times across all three TBAs). This demonstrates the importance of clarifying target behaviours across a clinical practice process, and the different kinds of barriers representing distinct behavioural drivers that might emerge. Furthermore, our findings highlight what these drivers might stem from or connect to through the additional information presented regarding overarching TDF themes. For example, ‘environmental context and resources’ was coded four times as a distinct barrier to ensuring appropriate patients are selected for genomic testing (TBA1): (a) *takes too long*; (b) *unable to join meetings*; (c) *lack of genetic counsellor support at offering stage (coded twice)*. However, the distinct domains fit into three separate overarching themes: (a) ‘beliefs about consequences’—*lack of understanding/appreciation of the value of testing*; (b) ‘beliefs about capabilities’—*confidence in ability to do genomic testing (coded twice)* and (c) ‘social influences’—*faith in ability and integrity of others to ensure appropriate patients are tested*.

Previous research has demonstrated the frequency of overlapping TDF domains, and some of the challenges this presents with specifying a domain which a particular barrier represents, as well as determining a corresponding BCT for designing an appropriate implementation strategy [17–19]. In providing both the context-based and hypothesised theoretical links between distinct and overarching barriers, plus the mechanistic links to intuitive- and theory-driven BCTs (see Figs. 2 and 3), the transparency of the likely behaviour change pathway from barrier to implementation strategy is enhanced. Furthermore, taking the influencing factors and psychosocial consequences of a barrier on other domains into account produced carefully considered (a) pathways between intuitively-derived strategies and their hypothesised mechanistic effects, and (b) theory-driven implementation strategy development by taking potential flow on effects into account. This approach may help to illuminate mechanistic effects of strategies that incorporate multiple BCTs, which have previously demonstrated greater impact on behaviour change than those that do not [59].

Using an approach whereby non-genetic medical specialists were asked about factors that help or hinder the implementation of genomics into clinical practice elicited 29 intuitive strategies, which demonstrated a solution for 23 out of the 32 identified barriers (72%). A total of 21 BCTs were represented within the 29 intuitive strategies on 49 occasions, yet they were found to align with theoretical recommendations on only ten (20%) of these occasions. This demonstrates that whilst clinicians are well positioned to develop logical solutions to address a given clinical problem, these solutions retrospectively align with underlying theory only part of the time. The extent of alignment varied both within and between BCTs used. For example, the BCT ‘conserving mental resources’ was used on seven occasions (across distinct domains and overarching themes), demonstrated links on three occasions (all with TDF domain ‘memory, attention, and decision processes’), non-links on two occasions (both with TDF domain ‘social and professional role and identity’), and for the remaining two occasions there was no evidence available. ‘Behavioural practice/rehearsal’ was used on three occasions and demonstrated links on two occasions with different TDF domains (‘skills’ and ‘beliefs about capabilities’), and for the remaining occasion there was no evidence available. Furthermore, the nature of the retrospectively mapped BCTs and the accompanying intuitive strategies were largely practical (e.g., related to continuously updated information provision, support from experienced colleagues, designing new systems/forms), whereas in many of the instances where no intuitive strategy was suggested and the research team were required to develop theory-driven strategies, the barriers were more complex (e.g., perceptions about organisational expectations to complete bureaucratic processes associated with feeding results back to patients; slow return of results to feed back to patients which manifested in expressions of frustration). In such circumstances, it may be that theory can be of greater benefit in the design of implementation strategies to address more complex barriers (e.g., belief systems, emotions).

**Fig. 6:**
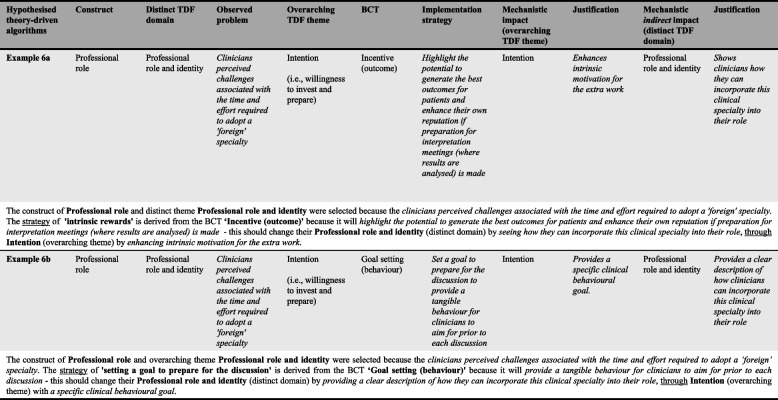
Practical application of theory-driven algorithm Note: Social and Professional Role and Identity currently has no BCTs with established mechanistic links recorded in the Theory and Techniques Tool and so it was not possible to use this approach to target this domain directly. However, the BCTs selected in this example aim to target "Intention" and, through increased motivation [via incentive (outcome) and goal setting (behaviour)], indirectly impact Social and Professional Role and Identity

When theory was explicitly used to guide the design of implementation strategies to address the remaining nine barriers for which no intuitive strategy was suggested, four of the 20 domain-matched BCTs selected were not identified as part of intuitively-derived strategies: *incentive (outcome)—*overarching TDF theme: ‘intentions’; *goal setting (behaviour)—*overarching TDF theme: ‘intentions’; *verbal persuasion about capability—*distinct TDF domain: ‘environmental context and resources; and *remove aversive stimulus—*distinct TDF domain: ‘environmental context and resources’. These BCTs may be perceived as more sophisticated than others (e.g., ‘demonstration of the behaviour’; ‘provide information on health consequences’, ‘social support’) and/or require more intricate application for the design of a specific implementation strategy to address a domain-matched barrier. As an example, Fig. 6 demonstrates two hypothesised behavioural pathways that could be generated using ‘incentive’ (*outcome*) and ‘goal setting’ *(behaviour)—*both utilised to address a distinct TDF domain via directly addressing an overarching TDF theme.

Whilst these findings and methodological advances may be insightful for the field of implementation science and help to inform health system evidence translation efforts, this research is not without limitations, which point towards avenues for future development and exploration. First, whilst we have proposed an algorithm to illustrate hypothesised behavioural pathways with mechanistic links, these steps have not yet been formally tested. Nonetheless, given the increasing calls for standardised reporting of intervention and implementation strategy design alongside program theory, this approach at the very least provides a step in the right direction. One alternative or indeed complementary process may be to incorporate the use of ‘implementation logic models’ to demonstrate intended MoAs and proposed causal pathways [60, 61]. It will also be important for future work to include other details in the pathways that align with guidance for specifying implementation strategies [62]. Second, the intuitively derived strategies were coded to BCTs based on the descriptive detail available from clinician interviews, but it is possible that these described strategies may not have captured the detail required in the BCT descriptions. Each intuitive strategy was subject to research team interpretation, and so there is arguably some variation on the extent to which alignment of intuitive strategies is completely accurate, and it is possible that some suggested strategies did not have sufficient detail to allow for mapping to be undertaken. Third, it would be difficult to ascertain clarity on the effects of intuitive strategies (whether they were theoretically aligned or not) over theory-driven strategies in this particular context given many were already implemented by healthcare professionals at the time interviews took place. Well designed, controlled implementation trials are needed to assess the difference between intuitively derived and theory-driven strategies on implementation success [40]. Fourth, the data was collected in 2018 and the evidence base for the effectiveness of BCTs for addressing specific TDF domains is continuously evolving [8, 9, 41]. The time taken to synthesise and standardise this data according to a behavioural pathway algorithm was extensive and undoubtedly too slow for health system implementation needs. Perhaps more automated approaches are needed to optimise the use of implementation data in a way that more efficiently supports those responsible for implementation.

## Conclusion

This work has advanced understanding of how to assess the extent to which healthcare professionals generate context-relevant theory-aligned implementation strategies to address identified barriers, and highlighted areas where theory might be most useful for implementation strategy design. All barriers have been mapped to the TDF, intuitive and theory-driven implementation strategies coded against BCTs, and standardised hypothesised behavioural pathways have been developed to make potential underlying theory explicit. The methods presented here have the potential to serve several purposes: (1) aid in efforts to collect and code intuitive strategies against theory, and subsequently assess alignment; (2) provide the foundations for building a body of evidence, both for genomics and across other clinical specialties, around (a) the value of clinician intuition for implementation strategy design, (b) narrowing down barriers for which the use of theory is most useful, or critical, to address; and (c) standardising implementation strategies designed intuitively and/or using theory to build more evidence for establishing patterns of cause and effect between changes in determinants and desired behaviour/practice change.

Coding intuitive strategies derived from clinician interviews and designing theory-driven strategies ‘from scratch’ required intensive effort from both the clinical and research teams. However, this body of work is imperative to support the development of a comprehensive theoretically informed tool to facilitate scale-up efforts of genomics. Such a tool could expedite development of theory-driven implementation strategies tailored to local barriers, particularly if it was embedded within an online knowledge learning system.

## Supplementary Information

**Additional file 1.** BCTTv1_PDF (behaviour change technique labels and definitions) [9].

**Additional file 2.** Genomics implementation barrier and strategy mapping and theory alignment (interview data coded to theoretical domains framework and behaviour change techniques, alignment of intuitive strategies against behaviour change techniques).

**Additional file 3.** TDF domains and BCTs counting exercise (counts of number of barriers, TDF domains represented, behaviour change techniques used, theoretical alignment of intuitive strategies).

**Additional file 4.** TIDieR-Checklist_completed.

## Data Availability

All data generated or analysed during this study are included in this published article [and its additional information files].

## Abbreviations

BCT: Behaviour Change Technique
MoA: Mechanism of Action
TBA: Target Behaviour Area
TDF: Theoretical Domains Framework
TIDieR: Template for Intervention Description and Replication
